# Online Sequential Projection Vector Machine with Adaptive Data Mean Update

**DOI:** 10.1155/2016/5197932

**Published:** 2016-04-07

**Authors:** Lin Chen, Ji-Ting Jia, Qiong Zhang, Wan-Yu Deng, Wei Wei

**Affiliations:** ^1^School of Computer, Xi'an University of Posts & Telecommunications, Xi'an 710121, China; ^2^School of Computer Science and Engineering, Xian University of Technology, Xi'an 710048, China

## Abstract

We propose a simple online learning algorithm especial for high-dimensional data. The algorithm is referred to as online sequential projection vector machine (OSPVM) which derives from projection vector machine and can learn from data in one-by-one or chunk-by-chunk mode. In OSPVM, data centering, dimension reduction, and neural network training are integrated seamlessly. In particular, the model parameters including (1) the projection vectors for dimension reduction, (2) the input weights, biases, and output weights, and (3) the number of hidden nodes can be updated simultaneously. Moreover, only one parameter, the number of hidden nodes, needs to be determined manually, and this makes it easy for use in real applications. Performance comparison was made on various high-dimensional classification problems for OSPVM against other fast online algorithms including budgeted stochastic gradient descent (BSGD) approach, adaptive multihyperplane machine (AMM), primal estimated subgradient solver (Pegasos), online sequential extreme learning machine (OSELM), and SVD + OSELM (feature selection based on SVD is performed before OSELM). The results obtained demonstrated the superior generalization performance and efficiency of the OSPVM.

## 1. Introduction

In many real applications, such as text mining, visual tracking, and dynamical interest perception, there are always two problems: (1) new data arriving sequentially and (2) the data which is in high-dimensional space. For the first problem, many online sequential algorithms have been proposed [1–13]. SGBP [1] is one of the main variants of BP for sequential learning applications in which the network parameters are learned iteratively on the basis of first-order information. Crammer and Lee [7] proposed a new family of online learning algorithms based upon constraining the velocity flow over a distribution of weight vectors. Hoi et al. [8] proposed an online multiple kernel classification algorithm which learns a kernel-based prediction function by selecting a subset of predefined kernel functions in an online learning fashion. Wang et al. [9] proposed a Fourier online gradient descent algorithm that applies the random Fourier features for approximating kernel functions. Zhao et al. [14] proposed a fast bounded online gradient descent algorithm for scalable kernel-based applications that aims to constrain the number of support vectors by a predefined budget. Zhang et al. [11] proposed an online kernel learning algorithm which measures the difficulty in correctly classifying a training example by the derivative of a smooth loss function and gave more chance to a difficult example to be a support vector than an easy one via a sampling scheme. Shalev-Shwartz et al. [12] proposed a simple and effective stochastic subgradient descent algorithm primal estimated subgradient solver (Pegasos) for solving the optimization problem cast by Support Vector Machines (SVMs). Wang et al. [13] proposed an adaptive multihyperplane machine (AMM) model that consists of a set of linear hyperplanes (weights), each assigned to one of the multiple classes and predicts based on the associated class of the weight that provides the largest prediction. Wang et al. [10] proposed a budgeted stochastic gradient descent (BSGD) approach for training SVMs which keeps the number of support vectors bounded during training through several budget maintenance strategies. OSELM [15] is a very fast sequential algorithm derived from batch extreme learning machine (ELM) [16] in which the input weights are randomly generated and the output weights are determined by incremental least square. The aforementioned algorithms have their own advantages, respectively, in solving online learning problems for new data. However, they all thought that data preprocessing is independent on the model online learning. Different to these approaches, we propose an online learning algorithm OSPVM (online sequential projection vector machine) based on batch-PVM which enjoys the properties of combining data preprocessing (data centering and dimension reduction) and the model learning as a total. In our earlier work we have proposed incremental PVM [17] which can learn PVM incrementally; however, it cannot update data mean automatically. Data mean update is very important for improving the generalized performance of OSPVM. When new samples arrive, if the data mean is not updated, the components (features) obtained by SVD/PCA will shift and degrade the generalized performance. The proposed OSPVM algorithm enjoys three prosperities: (1) the mean of data can be updated dynamically, (2) projection vectors can be updated incrementally to capture more useful features from new data, and (3) the number of hidden nodes can be adjusted adaptively to ensure enough learning capability.

The paper is organized as follows.  Section 2 gives a brief review of the batch-PVM.  Section 3 presents the derivation of OSPVM. Performance evaluation of OSPVM is shown in Section 4 based on the benchmark problems in different areas. Conclusions based on the study and experiments are made in Section 5.

## 2. Review of Projection Vector Machine

This section briefly reviews the batch-PVM developed by Deng et al. [18] to provide the necessary background for the development of OSPVM in Section 3. In order to make it easy to read, some symbols are defined:

(i) [**A**, **B**]: horizontal concatenation of matrix **A** and **B**;
(ii) [**A**; **B**]: vertical concatenation of matrix **A** and **B**;
(iii) $\bar{A}=\left(\sum_{i=1}^{n}x_{i}\right)/n$: mean vector of **A**;
(iv) 1_1×*n*_ = [1,1,…, 1]_1×*n*_.

### 2.1. Single Hidden Layer Feedforward Neural Network (SLFN)

For *n* arbitrary distinct samples **ℵ** = {(**x**_*i*_, **t**_*i*_)}_*i*=1_^*n*^, where **x**_*i*_ = [*x*_*i*1_, *x*_*i*2_,…, *x*_*im*_]^*T*^ ∈ **R** ^*m*^ and **t**_*i*_ = [*t*_*i*1_, *t*_*i*2_,…, *t*_*iℓ*_]^*T*^ ∈ **R** ^*ℓ*^, a standard SLFN with $\tilde{N}$ hidden nodes and activation function *g*(*x*) are mathematically modeled as

(1) ∑i=1N~βigixk=∑i=1N~βigiwi·xk+bi=ok,k=1,2,…,n,

where **w**_*i*_ = [*w*_*i*1_, *w*_*i*2_,…, *w*_*im*_]^*T*^ ∈ **R** ^*m*^ is the input weight vector connected with the *i*th hidden nodes and the input nodes, *b*_*i*_ ∈ **R** is the threshold of *i*th hidden nodes, and **β**_*i*_ = [*β*_*i*1_, *β*_*i*2_,…, *β*_*iℓ*_]^*T*^ ∈ **R** ^*ℓ*^ is the output weight vector connecting with the *i*th hidden nodes and the output nodes. **w**_*i*_ · **x**_*k*_ denotes the inner product of **w**_*i*_ and **x**_*k*_. If *b*_*i*_ is treated input weights and denoted as *w*_*i*(*m*+1)_, then **w**_*i*_ can be extended to **w**_*i*_ = [*w*_*i*1_, *w*_*i*2_,…, *w*_*im*_, *w*_*i*(*m*+1)_]^*T*^ ∈ **R** ^*m*+1^ and the sample **x**_*k*_ is extended to [**x**_*k*_; 1]. Equation (1) can be transformed as

(2) $$\begin{array}{c} \overset{\tilde{N}}{\underset{i=1}{\sum }}\beta_{i}g_{i}\left(\;w_{i}\cdot \left[\;x_{k};1\;\right]\;\right)=o_{k},\;k=1,2,\ldots ,n. \end{array}$$

The above *n* equations can be written compactly as

(3) $$\begin{array}{c} g\left(\;H_{in}\;\right)\beta =T, \end{array}$$

where

(4) gHingWX;1=gw1·x1;1⋯gwN~·x1;1⋮⋯⋮gw1·xk;1⋯gwN~·xk;1n×N~,

$W=[w_{i},w_{2},\ldots ,w_{\tilde{N}}{]}^{T}$, $\beta =[\beta_{1},\beta_{2},\ldots ,\beta_{\tilde{N}}{]}^{T}$, **X** = [**x**_1_,…, **x**_*n*_]^*T*^, and **T** = [**t**_1_,…, **t**_*n*_]^*T*^. To train an SLFN, one may wish to find specific **W**, **β** to minimize the following cost function:

(5) $$\begin{array}{c} \xi \left(\;W,\beta \;\right)=\left\|\;g\left(\;W\left[\;X,1\;\right]\;\right)\beta -T\;\right\|. \end{array}$$

Gradient-based learning algorithms [19] are generally used to search (**W**, **β**) by minimizing *ξ*(**W**, **β**), but they are time-consuming and maybe stop at a local minima. Extreme learning machine (ELM) [16, 20] randomly chooses input weights **W** and analytically determines the output weights **β** by* Moore-Penrose generalized inverse*. ELM can learn hundreds of times faster than gradient-based learning algorithms. But for the high-dimension and small-sample data, ELM will become unstable seriously especially when the data is sparse (there are many zero features). In order to tackle this problem, we have proposed batch projection vector machine (Batch-PVM) [18].

### 2.2. Batch Projection Vector Machine (Batch-PVM)

Batch-PVM combines SLFN together with SVD seamlessly, in which the input weights of SLFNs are calculated from SVD. Given data **X**, through data centralization and extension, the data is transformed as $\left[X-1_{n\times 1}\bar{X};1\right]$ and its low rank SVD is

(6) $$\begin{array}{c} \left[\;X-1_{n\times 1}\bar{X};1\;\right]\overset{svd}{\leftarrow }U_{d}\Lambda_{d}V_{d}^{T}, \end{array}$$

where *d* is the truncated rank, **U**_*d*_^*T*^ is the projection vectors by which the data $\left[X-1_{n\times 1}\bar{X};1\right]$ is mapped into low-dimension space:

(7) $$\begin{array}{c} U_{d}^{T}\left[\;X-1_{n\times 1}\bar{X};1\;\right]=\Lambda_{d}V_{d}^{T}. \end{array}$$

Since the role of input weights of SLFN can be treated as dimension reduction, thus they can be directly obtained by

(8) $$\begin{array}{c} W=U_{d}^{T}. \end{array}$$

Naturally, the number of hidden nodes is determined by

(9) $$\begin{array}{c} \tilde{N}=d. \end{array}$$

The problem becomes linear problem, and thus the output weights **β** can be obtained by

(10) βgHin†T=gX−1n×1X¯;1UdT=gΛdVdT†T.

The experimental results in many classification and regression problems show that Batch-PVM is faster and more accurate than the familiar two-stage methods in which dimension reduction and SLFN training are independent. The batch-PVM assumes that all the training data (samples) are available, but, in real applications, some training data has been accumulated but at the same time new data will arrive chunk-by-chunk or one-by-one (a special case of chunk). The batch-PVM has to be modified for this case so as to make it able to learn online sequentially [21, 22].

## 3. The Proposed Online Sequential Algorithm

The seamless combination of dimension reduction and SLFN training facilitates the design of sequential online learning. Once the SVD is updated for new samples, the dimension reduction projection matrix and all the parameters $\left\{W,\beta ,\tilde{N}\right\}$ of SLFN can be updated conveniently.

### 3.1. Data Mean and Projection Vectors Update

Assume that *n*_*a*_ training samples **ℵ**_*a*_ = {(**x**_*i*_, **t**_*i*_)}_*i*=1_^*n*_*a*_^ have been available so far, the inputs and targets are denoted as **A** = [**x**_1_, **x**_2_,…, **x**_*n*_*a*__]^*T*^ and **T**_*a*_ = [**t**_1_, **t**_2_,…, **t**_*n*_*a*__]^*T*^, respectively. By centralization (subtracting the mean of the inputs) and extension, the data can be transformed as

(11) $$\begin{array}{c} \hat{A}=\left[\;A-\bar{A}1_{1\times n_{a}};1\;\right]. \end{array}$$

The SVD of $\hat{A}$ with the truncated rank *r*_*a*_ is

(12) $$\begin{array}{c} \hat{A}\overset{svd}{\leftarrow }U_{r_{a}}\Lambda_{r_{a}}V_{r_{a}}^{T},\;r_{a}\ll m. \end{array}$$

Assume that *k*th chunk of data **ℵ**_*b*_ = {(**x**_*i*_, **t**_*i*_)}_*i*=1_^*n*_*b*_^ is presented where the new inputs and targets are denoted as **B**_*k*_ = [**x**_1_, **x**_2_,…, **x**_*n*_*b*__] and **T**_*b*_ = [**t**_1_, **t**_2_,…, **t**_*n*_*b*__], respectively, and the horizontal concatenation of **A** and **B**_*k*_ is denoted as **C** = [**A**, **B**_*k*_]_*m*×(*n*_*a*_+*n*_*b*_)_.

The update task is to get the new mean $\bar{C}$ and SVD of $\left[C-1_{(n_{a}+n_{b})\times 1}\bar{C};1\right]$; that is,

(13) $$\begin{array}{c} \left[\;C-1_{\left(n_{a}+n_{b}\right)\times 1}\bar{C};1\;\right]\overset{svd}{\leftarrow }U_{r_{c}}\Lambda_{r_{c}}V_{r_{c}}^{T}. \end{array}$$

There are many sophisticated algorithms that have been developed to efficiently update SVD as more data arrive [23]. However, most approaches assume that the sample mean is fixed when updating the eigenbasis or equivalently that the data is inherently zero-mean. This assumption does not hold in many applications. New samples will lead to the change of data mean and thus the mean needs to be recomputed before updating SVD. One approach proposed by Hall et al. [24] considered the change of the mean while updating SVD as one set of new data arrives. However the high computational cost is a bottleneck of this method applied to many applications. Here we will extend Sequential Karhunen-Loeve [25] algorithm to make it suitable for updating SVD efficiently with mean update simultaneously.

First we update the mean. The mean vector of **A** and **B**_*k*_ is $\bar{A}=\left(\sum_{i=1}^{n_{a}}x_{i}\right)/n_{a}$, ${\bar{B}}_{k}=\left(\sum_{i=1}^{n_{b}}x_{i}\right)/n_{b}$, so the mean vector of **C** is

(14) $$\begin{array}{c} \bar{C}=\frac{\sum_{i=1}^{n_{a}+n_{b}}x_{i}}{n_{a}+n_{b}}=\frac{\sum_{i=1}^{n_{a}}x_{i}+\sum_{i=1}^{n_{b}}x_{i}}{n_{a}+n_{b}}=\frac{n_{a}\bar{A}+n_{b}{\bar{B}}_{k}}{n_{a}+n_{b}}. \end{array}$$

It is not difficult to find that

(15) C−C¯11×na+nb;1=A−A¯11×na;1,Bk−A¯11×nb;1+A¯−C¯11×na+nb;0=A^,B^k+A¯−C¯11×na+nb;0,

where $\hat{A}=[A-\bar{A}1_{1\times n_{a}};1]$ and ${\hat{B}}_{k}=[B_{k}-\bar{A}1_{1\times n_{b}};1]$. Since the SVD of $\hat{A}$ has been known, this means that we can compute the SVD of $\left[\hat{A},{\hat{B}}_{k}\right]$ by incremental algorithm [18]:

(16) $$\begin{array}{c} \left[\;\hat{A},{\hat{B}}_{k}\;\right]=\hat{U}\hat{\Lambda }{\hat{V}}^{T}. \end{array}$$

Denote ${\dot{V}}^{T}={\hat{V}}^{T}-\bar{{\hat{V}}^{T}}1_{1\times (n_{a}+n_{b})}$; then we have ${\hat{V}}^{T}={\dot{V}}^{T}+\bar{{\hat{V}}^{T}}1_{1\times (n_{a}+n_{b})}$. Therefore,

(17) A^,B^k=U^Λ^V˙T+V^T¯11×na+nb=U^Λ^V˙T+U^Λ^V^T¯11×na+nb=U^Λ^V˙T+A^,B^k¯11×na+nb=U^Λ^V˙T+A−A¯11×na;1,Bk−A¯11×nb;1¯11×na+nb=U^Λ^V˙T+A,Bk¯−A¯,A¯¯;011×na+nb=U^Λ^V˙T+C¯−A¯11×na+nb;0.

Substituting it into (15), we have

(18) C−C¯11×na+nb;1U^Λ^V˙T+C¯−A¯11×na+nb;0+A¯−C¯11×na+nb;0=U^Λ^V˙T.

It is obvious that the SVD of $\left[C-1_{(n_{a}+n_{b})\times 1}\bar{C};1\right]$ can be calculated based on the SVD of $\hat{U}\hat{\Lambda }{\dot{V}}^{T}$. Perform QR-decomposition of $\dot{V}$,

(19) $$\begin{array}{c} \dot{V}=\dot{Q}\dot{R}. \end{array}$$

Substituting (19) into (18) we have

(20) $$\begin{array}{c} \left[\;C-\bar{C}1_{1\times \left(n_{a}+n_{b}\right)};1\;\right]=\left(\;\hat{U}\hat{\Lambda }{\dot{R}}^{T}\;\right){\dot{Q}}^{T}. \end{array}$$

Perform SVD on $\hat{U}\hat{\Lambda }{\dot{R}}^{T}$:

(21) $$\begin{array}{c} U_{f}\Lambda_{f}V_{f}^{T}=\hat{U}\hat{\Lambda }{\dot{R}}^{T}. \end{array}$$

Substituting (21) into (20) we get the SVD of $\left[C-\bar{C}1_{1\times (n_{a}+n_{b})};1\right]$:

(22) $$\begin{array}{c} C-\bar{C}1_{1\times \left(n_{a}+n_{b}\right)}=\underset{U}{\underset{︸}{U_{f}}}\underset{\Lambda }{\underset{︸}{\Lambda_{f}}}\underset{V^{T}}{\underset{︸}{{\left(V_{f}\dot{Q}\right)}^{T}}}. \end{array}$$

Go back to the SVD of $\left[\hat{A},{\hat{B}}_{k}\right]$. Let ${\tilde{B}}_{k}$ be component of ${\hat{B}}_{k}$ orthogonal to **U**_*r*_*a*__; that is,

(23) $$\begin{array}{c} {\tilde{B}}_{k}⟵\text{orth}\left(\;{\hat{B}}_{k}-U_{r_{a}}U_{r_{a}}^{T}{\hat{B}}_{k}\;\right). \end{array}$$

We can get the following partitioned form:

(24) $$\begin{array}{c} \left[\;\hat{A},{\hat{B}}_{k}\;\right]\overset{svd}{\leftarrow }\left[\;U_{r_{a}}{\tilde{B}}_{k}\;\right]\left[\begin{array}{cc} \Lambda_{r_{a}} & U_{r_{a}}^{T}{\hat{B}}_{k}\\ 0 & {\tilde{B}}_{k}^{T}{\hat{B}}_{k} \end{array}\right]\left[\begin{array}{cc} V_{r_{a}}^{T} & 0\\ 0 & I \end{array}\right]. \end{array}$$

Let $M=\left[\begin{array}{cc} \Lambda_{r_{a}} & U_{r_{a}}^{T}{\hat{B}}_{k}\\ 0 & {\tilde{B}}^{T}{\hat{B}}_{k} \end{array}\right]$. The SVD of **M** can be computed in constant time regardless of the following:

(25) $$\begin{array}{c} M\overset{svd}{\leftarrow }\tilde{U}\tilde{\Lambda }{\tilde{V}}^{T}. \end{array}$$

So we get the SVD of $\left[\hat{A},{\hat{B}}_{k}\right]$,

(26) $$\begin{array}{c} \left[\;\hat{A},{\hat{B}}_{k}\;\right]\overset{svd}{\leftarrow }\underset{\hat{U}}{\underset{︸}{\left(\left[U{\tilde{B}}_{k}\right]\tilde{U}\right)}}\underset{\hat{\Lambda }}{\overset{\sim }{\underset{︸}{\Lambda }}}\underset{{\hat{V}}^{T}}{\underset{︸}{\left({\tilde{V}}^{T}\left[\begin{array}{cc} V^{T} & 0\\ 0 & I \end{array}\right]\right)}}. \end{array}$$

### 3.2. Hidden Nodes Update Adaptively

The number of hidden nodes is very important for SLFN [6]. Too many hidden nodes lead to overfitting while too few hidden nodes might lead to insufficiency of learning capability. When new training samples are presented the hidden nodes should be added to ensure the SLFN model possesses enough learning capability. OP-ELM [26] ranked the hidden nodes by multiresponse sparse regression (MRSR) and then make the final decision over the appropriate number of nodes by Leave-One-Out (LOO) validation method. I-ELM [27] increase random hidden nodes one-by-one until the residual error is smaller than one given threshold value. EI-ELM [28] selected the optimized random hidden nodes from one random hidden nodes set before increasing hidden node one-by-one. C-ELM [29] associate each model term to a regularized parameter; as a result, insignificant ones are automatically penalized and unselected. Since, in PVM, the number of hidden nodes $\tilde{N}$ is equal to the target low rank *d* of SVD, we will adopt accumulation ratio of principle components to determine the number of nodes. The accumulation ratio is defined by [30] as follows:

(27) $$\begin{array}{c} \gamma \left(\;\tilde{N}\;\right)=\frac{\sum_{i=1}^{\tilde{N}}\sigma_{i}}{\sum_{i=1}^{r_{c}}\sigma_{i}}, \end{array}$$

where *σ*_*i*_ denotes the singular value constituting the singular value diagonal matrix Λ_*r*_*c*__ = Diag{*σ*_1_, *σ*_2_,…, *σ*_*r*_*c*__}, $\tilde{N}$ denotes the number of hidden nodes, and *r*_*c*_ is number of nonzero singular values. By choosing one proper value $\tilde{N}$ that makes $\gamma (\tilde{N})<\theta$ hold, where *θ* is a given threshold value, we can get the new number of hidden nodes. The new input weights can be updated by

(28) $$\begin{array}{c} W=U_{{\tilde{N}}_{new}}. \end{array}$$

The output weight is updated by

(29) $$\begin{array}{c} \beta =g{\left(\;\Lambda_{{\tilde{N}}_{new}}V_{{\tilde{N}}_{new}}^{T}\;\right)}^{†}\left[\begin{array}{c} T_{a}\\ T_{b} \end{array}\right]. \end{array}$$

The algorithm can be summarized as Algorithm 1.

### 3.3. Theoretical Analysis: OSPVM versus OSELM

It is very difficult to prove OSPVM is better than OSELM strictly. So here we just give some theoretical analysis about OSPVM being better than OSELM from feature learning opinion.

As discussed in literature [31], minimizing reconstruction error is one very important condition to learn useful features. Reconstruction error of OSELM can be written as

(30) $$\begin{array}{c} E_{OSELM}={\left\|\;X-XW_{rand}W_{rand}^{†}\;\right\|}_{F}^{2}, \end{array}$$

where **X** ∈ **R** ^*n*×*m*^ is inputs (*n* is the number of instances and *m* is the dimensionality of data), **X**_rand_ ∈ **R** ^*m*×*N*^ (*N* is the number of hidden nodes) is input weights which are random values, and ‖·‖_*F*_^2^ is Frobenius norm. Reconstruction error of OSPVM can be written as

(31) $$\begin{array}{c} E_{OSPVM}={\left\|\;X-XW_{SVD}W_{SVD}^{T}\;\right\|}_{F}^{2}; \end{array}$$

**W**_SVD_ is input weights and obtained by singular value decomposition (SVD) as follows:

(32)  UN~SN~VN~T←N~-rank  SVDXs.t. UN~TUN~=I, VN~TVN~=I, WSVD⟵VN~.

Substituting $W_{SVD}\leftarrow V_{\tilde{N}}$ into *E*_OSPVM_, we have

(33) EOSPVMX−XVN~VN~TF2=X−UN~SN~VN~TVN~VN~TF2=X−UN~SN~VN~TVN~VN~TF2=X−UN~SN~IVN~TF2=X−UN~SN~VN~TF2;

${\left\|X-U_{\tilde{N}}S_{\tilde{N}}V_{\tilde{N}}^{T}\right\|}_{F}^{2}$ is the error of optimized rank-$\tilde{N}$ approximation of **X**; that is, ${\left\|X-U_{\tilde{N}}S_{\tilde{N}}V_{\tilde{N}}^{T}\right\|}_{F}^{2}$ is the minima of reconstruction error with rank $\tilde{N}$. Therefore, the reconstruction error *E*_OSELM_ of OSELM must be larger than that of OSPVM: *E*_OSELM_ > *E*_OSPVM_. In summary, when OSELM and OSPVM are with the same number of hidden nodes $\tilde{N}\ll n$, *E*_OSPVM_ is always smaller than *E*_OSELM_. Another condition to obtain better generalization performance is to make the hidden nodes $\tilde{N}$ as few as possible (Occam's Razor theory). Considering these two conditions, we can get the inferences: (1) when OSPVM and OSELM are with the same number of hidden nodes and satisfying *N* ≪ *n*, the reconstruction error of OSPVM is smaller than OSELM (*E*_OSPVM_ < *E*_OSELM_). This will help OSPVM to obtain better generalization performance in general, and (2) for the same reconstruction error OSPVM always needs less hidden nodes than OSELM. According to Occam's Razor theory, OSPVM will produce better generalization performance than OSELM with less hidden nodes.

Next, we briefly explain why OSPVM is better than SVD + OSELM in generalization performance in most cases. Similar to OSPVM, SVD + OSELM represents the data by SVD to obtain more useful features. However, SVD + OSELM discards the projection vectors obtained by SVD and still uses randomly values as input weights. In contrast, OSPVM uses the resulted projection vectors as input weights and thus can avoid the instability of random weights. So OSPVM can produce better generalization performance than SVD + ELM in most cases.

## 4. Performance Evaluation

### 4.1. Datasets and Experimental Settings

We select OSELM, BSGD, AMM, and Pegasos to compare with OSPVM on various UCI benchmark problems as shown in Table 1. For fair comparison, the feature selection by SVD is first conducted before these algorithms. The number of reduced dimensions *d* and the number of hidden nodes $\tilde{N}$ are both gradually increased by an interval of 5 and the nearly optimal combinations $\left(d,\tilde{N}\right)$ are selected by cross-validation method. OSELM code is downloaded from ELM homepage (http://www.ntu.edu.sg/home/egbhuang/). BSGD, AMM, and Pegasos are downloaded from the BudgetedSVM website (http://www.dabi.temple.edu/budgetedsvm/). OSPVM and SVD + OSELM are implemented by ourselves. For OSELM and Batch-PVM, the number of hidden nodes is gradually increased by an interval of 5 and the nearly optimal one is then selected by cross-validation method. For OSPVM, the accumulation rate threshold *θ* is chosen in the range of [0.95,0.99] by cross-validation method for every especial application. The activation functions for OSELM, OSPVM, SVD + OSELM, and Batch-PVM are all set as sigmoid function *g*(*x*) = 1/(1 + *e* ^−*x*^). For BSGD we set the kernel as Gaussian kernel *𝒦*(**x**_*i*_, **x**_*j*_) = exp⁡(−(1/*σ*)‖**x**_*i*_ − **x**_*j*_‖^2^), the budget maintenance strategy is set as “merging” which is more accurate than another alternate “removing,” and the number of budgeted support vectors is determined by cross-validation method. For AMM, the limit on the number of weights per class in AMM is determined by cross-validation method, and the learning rate is set to 0.0001. All the simulations are running in MATLAB 7, Pentium i7 920@2.67 GHZ CPU, and 6 G RAM environment. Average results of 20 trials of simulations for each fixed size of SLFN are obtained and then finally the best performance including training accuracy, testing accuracy, training time, testing time, and *t*-test is reported in this paper. *t*-test [32] is used to evaluate the performance difference of the algorithms. Denoting testing accuracies on the five datasets of *i*th algorithm as **a**_*i*_ = *a*_*i*,1_, *a*_*i*,2_,…, *a*_*i*,5_, *t* value can be computed as follows:

(34) $$\begin{array}{c} t=\frac{{\bar{a}}_{i}-{\bar{a}}_{j}}{\sqrt{v_{i}^{2}/n_{i}+v_{j}^{2}/n_{j}}}, \end{array}$$

where ${\bar{a}}_{i}$ and ${\bar{a}}_{j}$ denote mean value of **a**_*i*_ and **a**_*j*_, *v*_*i*_^2^ and *v*_*j*_^2^ represent the variance of **a**_*i*_ and **a**_*j*_, and *n*_*i*_ and *n*_*j*_ denote the number of datasets (here *n*_*i*_ = *n*_*j*_ = 5). By checking *t*-table, we can obtain the significant level *p*. Notice that the smaller the *p* value the more significant the difference.

OSPVM is first compared with Bach-PVM, BSGD, AMM, and Pegasos in this section. The number of hidden nodes, training time, testing time, training accuracy, and testing accuracy are reported in Table 2. The *t*-test results including *t* value and significant level *p* are summarized in Table 3. We can find from Table 2 that OSPVM can achieve nearly the same generalization to Batch-PVM while the training time is longer than Batch-PVM. The 16-by-16 mode is faster than one-by-one. Taking “Face” dataset as an example, the training time of OSELM is about 1.5 seconds and 13.07 seconds in 16-by-16 and 1-by-1 model, respectively. The reason lies in the fact that the bigger the chunk size, the fewer the update frequency. Batch-PVM just needs 0.46 seconds for “Face” dataset. In fact, Batch-PVM is one extreme case that initial data is entire data and does not need any update. For new samples, OSPVM can learn incrementally while Batch-PVM has to be retrained from the start. Taking “Face” dataset as an example, the average updating time of OSPVM for every sample is around 1.5/200 = 0.0075 seconds, while, for Batch-PVM, since it has to be retrained from the start, the updating time for every sample will be about 0.460 seconds. OSPVM is much faster than Batch-PVM in updating time for each sample. Table 2 also reported the results of considered algorithms BSGD, AMM, and Pegasos. The training time of BSGD, AMM, and Pegasos consists of the costs of dimension reduction and model training. From Tables 2 and 3 we can find that OSPVM can obtain competitive generalization performance in comparison to BSGD with *t* = 0.183 and *p* > 0.1 and significantly better than AMM (*t* = 4.141 and 0.01 > *p* > 0.001) and Pegasos (*t* = 2.267 and 0.1 > *p* > 0.05) while taking shorter training time. Still taking “Face” dataset as an example, BSGD, AMM, and Pegasos need 1.542, 1.99, and 1.530 seconds to obtain 91.63%, 88.75%, and 86.38% testing accuracy while OSPVM takes 1.50 seconds for 92.87% accuracy.

### 4.2. One-by-One

In this section we will compare OSPVM, OSELM, and SVD + OSELM in one-by-one case. Their training and testing accuracy are reported in Table 4, *t* values are shown in Table 6 and training time and testing time are reported in Table 5. As observed from Tables 4 and 5, although OSELM can learn at the fastest speed, OSPVM can produce better generalization performance than OSELM with *t* = 0.950 and *p* > 0.1. OSPVM obtained improved performance in most cases compared to SVD + OSELM while saving training time. Taking “Face” dataset as an example, SVD + OSELM takes 22.40 s to produce 91.0% accuracy while OSPVM takes 13.07 s to reach 91.2% accuracy. The reason lies in the fact that OSPVM can learn useful features similar to SVD + ELM and remove the redundancy between dimension reduction and neural network training. For SVD + OSELM, two control parameters including target dimensions and the number of hidden nodes need to be tuned, while for OSPVM only one parameter needs to be determined. This will make OSPVM more simple to determine parameter settings and more convenient for usage in real applications than SVD + OSELM. As shown in Table 7 where the hidden nodes and target dimension are reported, OSPVM needs less hidden nodes than OSELM and SVD + OSELM. This means that OSPVM can achieve better responding ability than other algorithms.

### 4.3. Chunk-by-Chunk

The performance of OSPVM, SVD + OSPVM, and OS-ELM in chunk-by-chunk mode (here we select 16-by-16 as an example) is reported in Tables 8, 9, 10, and 11. The results are similar to one-by-one model.  Table 9 shows that OSPVM needs longer training time than OSELM but shorter training time than SVD + OSELM. Tables 8, 10, and 11 show that OSPVM obtained better generalization performance and more compact structure than OSELM and SVD + OSELM in most cases. This means that OSPVM can improve the stability of OSELM in solving small-sample and high-dimensional problems and inherits the advantage of OSELM in aspect of learning efficiency.

### 4.4. Adaptive Increase of the Number of Hidden Nodes

**Figure 1:**
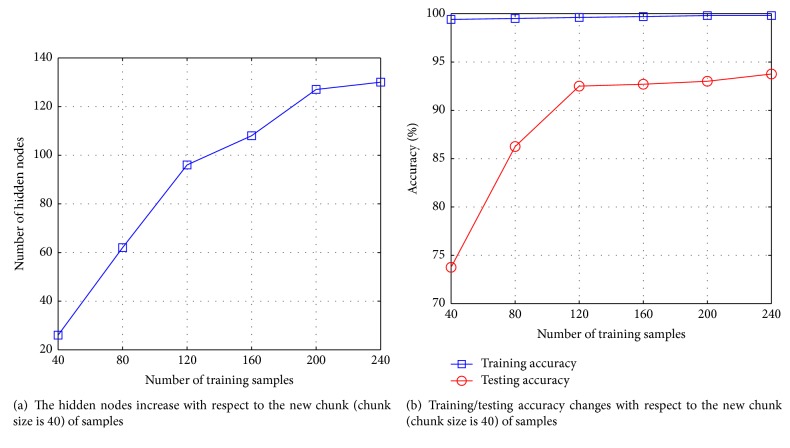
Adaptive model updating with respect to new samples (on Face dataset).

Figure 1(a) shows the curve of hidden nodes changing with increase of training samples. We can find that the hidden nodes of OSPVM grow adaptively when the new samples (chunk size is 40) are presented.  Figure 1(b) shows the curve of training accuracy and testing accuracy change with increase of the samples. We can observe that the cover capability (training accuracy) and generalized performance (testing accuracy) of the model always remain stable.

### 4.5. Equivalence of OSPVM and PVM

Data mean update together with projection vectors update is to ensure the obtained OSPVM is an accurate model which is equivalent to PVM rather than an approximation (if there is no data mean update, an approximate model would be obtained). This means that if having the same parameter setting (same number of hidden nodes, same training and testing splits, etc.), OSPVM and PVM would obtain the same performance (training accuracy and testing accuracy). To verify the equivalence of them, we run these two algorithms at the same setting on the benchmarks. From the results shown in Table 12, it can be found that OSPVM will obtain the same training accuracy and testing accuracy as PVM. This illustrates from experimental aspect that OSPVM is equivalent to PVM instead of an approximation and thus can obtain the same generalized ability.

### 4.6. The Influence of Mean Update to Generalized Performance of OSPVM

**Figure 2:**
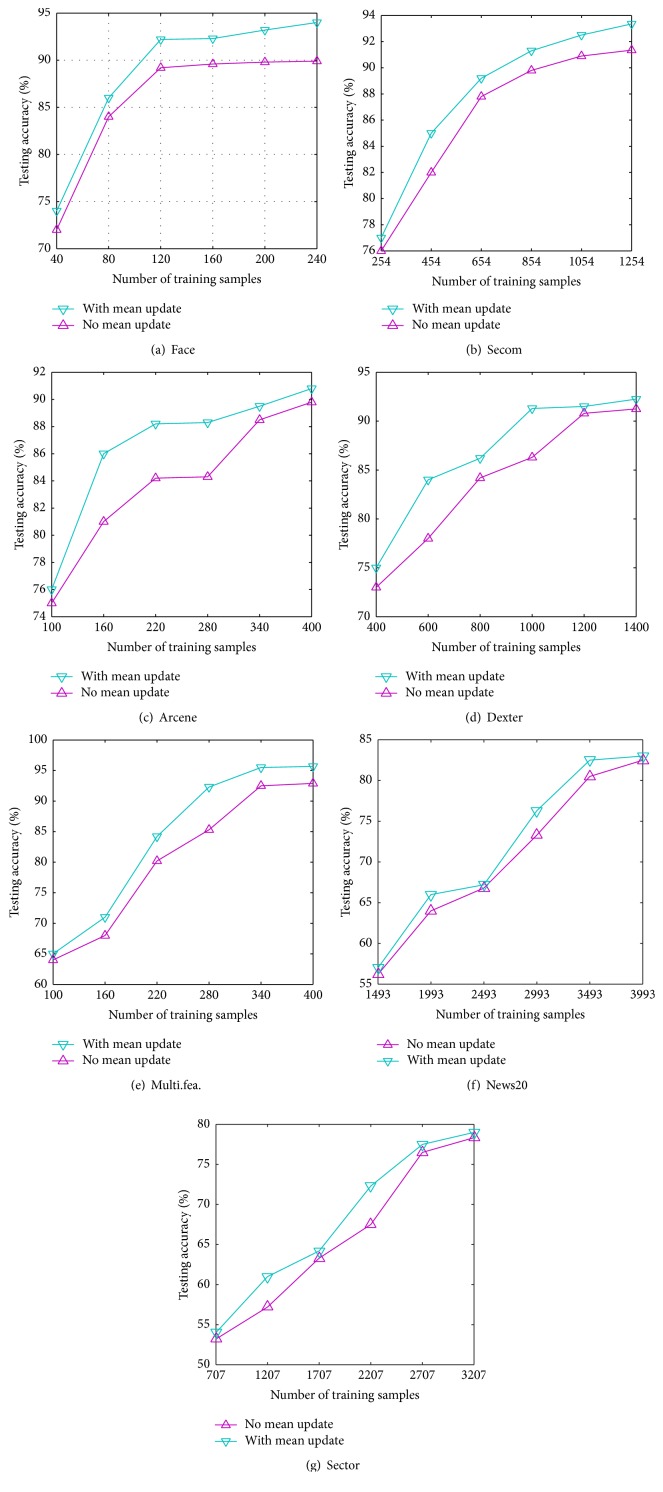
The influence of mean update to OSPVM.

To display the influence of the mean update to the generalized performance of OSPVM, we run OSPVM with two different settings, respectively, that is, “*with mean update*” and “*no mean update,*” on the same datasets including  Face, Secom, Arcene, Dexter, and Multi.fea. For “*with mean update*” setting, the data is centralized to mean and dynamically adjusted as well when the subsequent chunk of data arrives. The variation curves of the testing accuracy with respect to the chunk of training data under these two different settings are illustrated in Figure 2 (labeled as “*with mean update*” and “*no mean update,*” resp.). It can be found that, on each dataset, OSPVM* with mean update* always obtains better generalized performance than* no mean update*. Take Face dataset as an example, on the first 40 training samples, OSPVM* with mean update* attains 73.5% in terms of testing accuracy while “*no mean update*” attains 72.3%. Along with the arrival of the subsequent training data, OSPVM* with mean update* is also always superior to* no mean update*. In time of the last chunk of data arrival, the obtained testing accuracy “*with mean update*” reaches 94% while “*no mean update*” reaches 90%. From the point of view of theoretical analysis, the performance improvement is possibly due to two aspects:

- From principle component analysis perspective, the useful features are those directions with maximum variance [33]. In order to capture these directions, the data should be firstly centralized because, if there is no centralization, the first obtained direction which is from the origin to the centre will be shifted and the successive directions are also shifted consequently.
- On the other hand, from multivariate probability distribution perspective [34], the datasets are usually treated as a multivariate Gaussian distribution that is represented as the amount of the mean plus the variation along the principal vectors. By centering the data to the mean, the variational component of the data can be cancelled out and thus capture purely variational component of the data.

These experimental and theoretical analyses show that* mean update* has important positive influence to the generalized performance of OSPVM. With help of* mean update*, OSPVM can process dynamical data more adaptively and effectively.

## 5. Conclusion and Future Work

In this paper, an effective online sequential learning algorithm (OSPVM) has been proposed for high-dimensional and no-stationary data. Data mean, projection vectors, and neural network model can be updated simultaneously by one time pass of new samples. The algorithm can handle the new data arriving by one-by-one and chunk-by-chunk. Apart from setting the threshold value of accumulation ratio, no other parameter needs to be determined. Performance of OSPVM including training time and generalized performance is compared with some several typical online learning algorithms on real world benchmark problems. The results show that OSPVM can produce better generalization performance with more compact network structure than other algorithms in most cases. In our next work, we would further study how to improve computational efficiency to make it suitable for large data analytic. Additionally we would study more smart method to determine the threshold value of accumulation ratio adaptively.

## Figures and Tables

**Algorithm 1:**
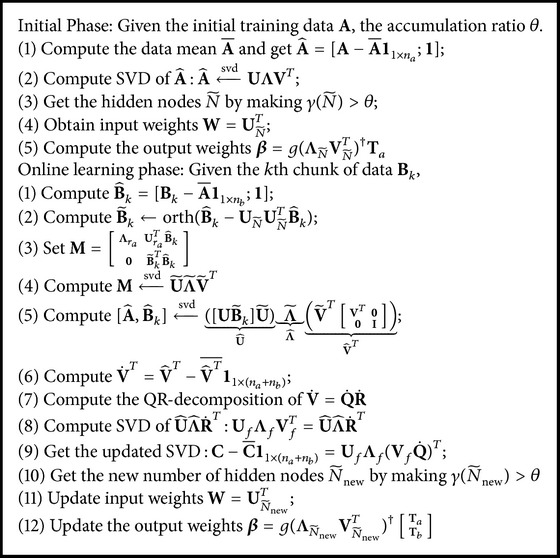
OSPVM algorithm.

**Table 1:** The specifications of the benchmark problems.

Dataset	#Training set	#Testing set	#Attributes	#Classes
Face	200	200	1600	10
Secom	1254	313	590	2
Arcene	400	500	10000	2
Dexter	1400	1200	20000	2
Multi.fea.	400	1600	650	10
News20	3993	15935	62061	20
Sector	3207	6412	55197	105

**Table 2:** Comparison of OSPVM, Batch-PVM, BSGD, AMM, and Pegasos.

Dataset	Algorithms	Nodes (θ)	Training time (s)	Testing time (s)	Training accuracy	Testing accuracy
Face	OSPVM (40, 16-by-16)	51 (0.96)	1.50 s	0.0004 s	99.89%	92.87%
	OSPVM (40, 1-by-1)	43 (0.99)	13.07 s	0.0005 s	99.20%	91.20%
	Batch-PVM	65	0.460 s	0.0005 s	99.81%	92.30%
	SVD + BSGD [10]	200	1.542 s	0.0835 s	99.92%	91.63%
	SVD + AMM Online [13]	200	1.990 s	0.0300 s	99.82%	88.75%
	SVD + Pegasos [12]	—	1.530 s	0.0240 s	99.11%	86.38%

Secom	OSPVM (40, 16-by-16)	61 (0.96)	1.67 s	0.007 s	94.08%	93.14%
	OSPVM (40, 1-by-1)	16 (0.96)	4.01 s	0.0004 s	94.14%	93.3%
	Batch-PVM	60	0.525 s	0.0073 s	93.37%	93.35%
	SVD + BSGD	100	1.801 s	0.0083 s	95.12%	93.13%
	SVD + AMM Online	100	12.19 s	0.031 s	94.11%	87.87%
	SVD + Pegasos	—	1.660 s	0.026 s	93.16%	89.12%

Arcene	OSPVM (40, 16-by-16)	106 (0.96)	61.17 s	0.0005 s	95.88%	90.50%
	OSPVM (40, 1-by-1)	39 (0.96)	130.6 s	0.0004 s	93.5%	86.7%
	Batch-PVM	85	5.06 s	0.00038 s	94.63%	90.80%
	SVD + BSGD	200	65.22 s	0.0335 s	95.92%	90.43%
	SVD + AMM Online	200	81.69 s	0.06 s	94.89%	87.75%
	SVD + Pegasos	—	56.41 s	0.044 s	94.42%	86.31%

Dexter	OSPVM (40, 16-by-16)	176 (0.96)	131.1 s	0.004 s	97.88%	92.25%
	OSPVM (40, 1-by-1)	86 (0.96)	619.3 s	0.004 s	96.0%	91.20%
	Batch-PVM	160	10.36 s	0.005 s	98.38%	91.25%
	SVD + BSGD	200	148.54 s	0.003 s	97.98%	92.63%
	SVD + AMM Online	200	178.19 s	0.003 s	96.81%	89.95%
	SVD + Pegasos	—	119.40 s	0.004 s	95.87%	87.36%

Multi.fea.	OSPVM (40, 16-by-16)	55 (0.96)	4.93 s	0.0053 s	98.16%	94.40%
	OSPVM (40, 1-by-1)	38 (0.96)	13.4 s	0.0047 s	96.6%	93.4%
	Batch-PVM	160	1.83 s	0.0192 s	99.98%	95.67%
	SVD + BSGD	200	5.54 s	0.0095 s	98.42%	94.63%
	SVD + AMM Online	200	10.79 s	0.03 s	99.82%	92.15%
	SVD + Pegasos	—	4.46 s	0.034 s	99.82%	91.88%

News20	OSPVM (40, 16-by-16)	1110 (0.96)	1283 s	19.8 s	85.26%	83.10%
	OSPVM (40, 1-by-1)	1100 (0.96)	1949 s	19.9 s	85.6%	83.14%
	Batch-PVM	1000	1060 s	19.2 s	84.89%	83.12%
	SVD + BSGD	1200	2289 s	18.6 s	83.52%	82.33%
	SVD + AMM Online	1200	2679 s	21.3 s	83.83%	82.25%
	SVD + Pegasos	—	1679 s	19.2 s	83.22%	81.81%

Sector	OSPVM (40, 16-by-16)	130 (0.96)	10.12 s	0.20 s	88.86%	78.40%
	OSPVM (40, 1-by-1)	150 (0.96)	18.4 s	0.21 s	86.6%	79.04%
	Batch-PVM	160	2.13 s	0.21 s	87.98%	79.01%
	SVD + BSGD	200	7.53 s	0.34 s	87.44%	76.68%
	SVD + AMM Online	200	12.69 s	0.33 s	86.81%	76.65%
	SVD + Pegasos	—	6.45 s	0.34 s	86.12%	75.88%

Note: since OSPVM is equivalent to PVM rather than an approximation,  if it has the same experimental setting (same number of hidden nodes and same training and testing splits), OSPVM and PVM would obtain the same performance (training accuracy and testing accuracy).

**Table 3:** *t* value and significant level of OSPVM versus BSGD, AMM, and Pegasos.

	SVD + BSGD (88.78%)	SVD + AMM (86.47%)	SVD + Pegasos (85.53%)
OSPVM (16-by-16) (89.23%)	*t* = 0.183, *p* > 0.1	*t* = 4.141, 0.01 > *p* > 0.001	*t* = 2.267, 0.1 > *p* > 0.05
OSPVM (1-by-1) (88.18%)	*t* = 1.434, *p* > 0.1	*t* = 1.958, 0.1 > *p* > 0.05	*t* = 2.932, 0.05 > *p* > 0.01

**Table 4:** Comparison of *training* and *testing accuracy* (in %) (one-by-one).

Dataset	SVD + OSELM		OSPVM		OSELM	
	Training accuracy	Testing accuracy	Training accuracy	Testing accuracy	Training accuracy	Testing accuracy
Face	99.8%	91.0%	99.2%	91.2%	98.1%	88.5%
Secom	93.3%	93.0%	94.14%	93.3%	93.2%	92.4%
Arcene	93.0%	83.0%	93.5%	86.7%	86.1%	81.1%
Dexter	95.7%	91.4%	96.0%	91.2%	75.6%	86.2%
Multi.fea.	99.0%	92.8%	96.6%	93.4%	96.5%	93.0%
News20	85.12%	82.9%	85.6%	83.14%	85.5%	83.0%
Sector	89.11%	77.8%	88.6%	79.04%	89.1%	78.1%

**Table 5:** Comparison of *training *and* testing time* (in seconds) (one-by-one).

Dataset	SVD + OSELM		OSPVM		OSELM	
	Training time	Testing time	Training time	Testing time	Training time	Testing time
Face	22.40 s	0.0006 s	13.07 s	0.0005 s	0.156 s	0.035 s
Secom	7.809 s	0.015 s	4.010 s	0.0004 s	0.346 s	0.029 s
Arcene	131.5 s	0.0004 s	130.6 s	0.0004 s	4.390 s	0.337 s
Dexter	619.3 s	0.001 s	519.8 s	0.0006 s	9.218 s	0.281 s
Multi.fea.	13.51 s	0.042 s	13.40 s	0.0167 s	1.164 s	0.097 s
News20	1987 s	19.1 s	1949 s	19.9 s	611 s	19.7 s
Sector	18.79 s	0.22 s	18.4 s	0.21 s	3.34 s	0.39 s

**Table 6:** *t* value and significant level of OSPVM versus SVD + ELM (1-by-1) and OSELM (1-by-1).

	SVD + OSELM (1-by-1) (86.73%)	OSELM (1-by-1) (86.04%)
OSPVM (1-by-1) (88.18%)	*t* = 0.7858, *p* > 0.1	*t* = 0.950, *p* > 0.1

**Table 7:** The number of hidden nodes (one-by-one).

Dataset	SVD + OSELM		OSPVM	OSELM
	#Target dimensions	#Hidden nodes		
Face	43	60	43	72
Secom	16	60	16	72
Arcene	39	110	39	160
Dexter	86	170	86	200
Multi.fea.	38	180	38	160
News20	780	1200	1100	1200
Sector	90	150	150	250

**Table 8:** Comparison of training and testing accuracy (in %) (16-by-16).

Dataset	SVD + OSELM		OSPVM		OSELM	
	Training accuracy	Testing accuracy	Training accuracy	Testing accuracy	Training accuracy	Testing accuracy
Face	99.82%	91.50%	99.89%	92.07%	98.22%	87.7%
Secom	93.34%	93.36%	94.08%	93.14%	93.32%	93.3%
Arcene	93.63%	89.90%	95.88%	90.50%	94.1%	89.7%
Dexter	89.75%	91.90%	97.88%	92.25%	72.6%	88.5%
Multi.fea.	99.48%	93.49%	98.16%	94.40%	96.78%	93.0%
News20	86.11%	83.09%	85.26%	83.10%	85.24%	81.0%
Sector	89.18%	78.19%	88.86%	78.40%	88.78%	76.20%

**Table 9:** Comparison of training and testing time (in seconds) (16-by-16).

Dataset	SVD + OSELM		OSPVM		OSELM	
	Training time	Testing time	Training time	Testing time	Training time	Testing time
Face	1.58 s	0.0005 s	1.5 s	0.0004 s	0.078 s	0.035 s
Secom	1.85 s	0.018 s	1.67 s	0.007 s	0.061 s	0.040 s
Arcene	61.4 s	0.0008 s	61.17 s	0.0005 s	2.03 s	0.55 s
Dexter	135.7 s	0.0006 s	131.1 s	0.0004 s	4.88 s	0.718 s
Multi.fea.	5.15 s	0.0218 s	4.93 s	0.0053 s	0.26 s	0.098 s
News20	1283 s	19.8 s	1283 s	19.8 s	0.26 s	0.098 s
Sector	10.7	0.21 s	10.12	0.20 s	5.26 s	0.38 s

**Table 10:** *t* value and significant level (*p*) of OSPVM versus SVD + ELM (16-by-16) and OSELM (16-by-16).

	SVD + OSELM (16-by-16) (88.7%)	OSELM (16-by-16) (87.05%)
OSPVM (16-by-16) (89.23%)	*t* = 0.7900, *p* > 0.1	*t* = 2.45, 0.05 > *p* > 0.01

**Table 11:** The number of hidden nodes (16-by-16).

Dataset	SVD + OSELM		OSPVM	OSELM
	#Target dimensions	#Hidden nodes		
Face	62	60	62	72
Secom	54	60	54	72
Arcene	110	110	106	300
Dexter	176	170	176	400
Multi.fea.	55	180	55	160
News20	780	1200	1100	1200
Sector	90	150	150	250

**Table 12:** Training and testing accuracy of PVM and OSPVM with the same hidden nodes.

Dataset	Algorithms	#Hidden nodes	Training accuracy	Testing accuracy
Face	OSPVM (1-by-1 and 16-by-16)	65	99.81%	92.30%
	Batch-PVM	65	99.81%	92.30%

Secom	OSPVM (1-by-1 and 16-by-16)	60	93.37%	93.35%
	Batch-PVM	60	93.37%	93.35%

Arcene	OSPVM (1-by-1 and 16-by-16)	85	94.63%	90.80%
	Batch-PVM	85	94.63%	90.80%

Dexter	OSPVM (1-by-1 and 16-by-16)	160	98.38%	91.25%
	Batch-PVM	160	98.38%	91.25%

Multi.fea.	OSPVM (1-by-1 and 16-by-16)	160	99.98%	95.67%
	Batch-PVM	160	99.98%	95.67%

News20	OSPVM (1-by-1 and 16-by-16)	1000	84.89%	83.12%
	Batch-PVM	1000	84.89%	83.12%

Sector	OSPVM (1-by-1 and 16-by-16)	160	87.98%	79.01%
	Batch-PVM	160	87.98%	79.01%
